# Meetings of Societies

**Published:** 1927

**Authors:** 


### Meetings of Societies.

### Bristol Medico-Chirurgical Society.

*April 13th,* 1927.

Dr. A. L. Flemming, President, in the Chair.

Dr. R. S. S. Statham read a paper on "**Dysmenorrhœa,**" reported on page 187.

The President asked whether Dr. Statham found that the incidence of dysmenorrhœa reflected the recent diminution in chlorosis.

Dr. R. C. Leonard related his factory experiences. He was constantly being called to the Ambulance Room to deal with severe attacks. He found acetanilid gave great relief ; benzyl benzoate he had also used. What was the maximum dose of the latter ?

Dr. R. Burges asked whether the sufferers from severe spasmodic dysmenorrhœa were likely to become pregnant, and if so, did the pregnancy lead to relief.

Dr. R. G. Gordon discussed psycho-therapeutic methods. He considered that psycho-analysis was of very little value in these cases, and hypnosis of none. On the contrary, he believed treatment along these lines was harmful and even dangerous.

Dr. R. S. Carey asked what dose of thyroid gland the lecturer considered advisable. He had himself been giving three grains a day for spasmodic dysmenorrhœa.

Colonel A. E. J. Lister said that there was a definite connection between the state of the genital glands and the lens of the eye. Was the "false cataract" of women, he asked, due to some toxin from the genital tract ? It was most frequent in the sufferers from genital trouble. Why did a woman's "eyes trouble her" during the menstrual period? He suggested that during this phase a toxin was produced and poured into the circulation.

DR. G. T. MYLES suggested that physical exercise played a large part in the elimination of dysmenorrhœa from schools.

MR. J. LACY FIRTH asked whether Dr. Statham found many patients developed dysmenorrhœa of the congestive type after appendix operations. He considered the sequence rare. How many laparotomies were nugatory?

MR. W. A. JACKMAN considered that the abolition of corsets had much to do with the recent improvement in dysmenorrhœa.

DR. J. H. C. EGLINTON stated that he had been employing heliotherapy in the treatment of this condition with encouraging results.

DR. STATHAM in reply said that the recent improvement in living conditions generally for women, including clothes, food and exercise, had much to do with the diminution of dysmenorrhœa as well as chlorosis. Spasmodic dysmenorrhœa lessened the chances of pregnancy, but usually disappeared after the latter occurred. He believed that in cases of *cataractus cœruleus*, the cause of which was unknown, the calcium metabolism was deranged. It was however certain that a toxin was responsible for congestive dysmenorrhœa. For the latter condition, unless there was a demonstrable and palpable lesion, he tried to avoid operation.

Benzyl benzoate was uncertain in its action; he employed ♏xx of 20 per cent. solution *quartis horis*. As to thyroid, he increased the dose gradually until toxic symptoms appeared. Morphin and heroin should at all costs be avoided in dysmenorrhœa; their use in this condition had often led to addiction; aspirin was safer, as the consequence of addiction were not serious.

